# Retroperitoneal-necrotizing fasciitis due to chronic pyelonephritis

**DOI:** 10.4103/0974-2700.70763

**Published:** 2010

**Authors:** J E EL Ammari, M Ahssaini, M J EL Fassi, M H Farih

**Affiliations:** Urology Department, Universitary Hospital Center Hassan II - FES, Morocco

**Figure 1:**
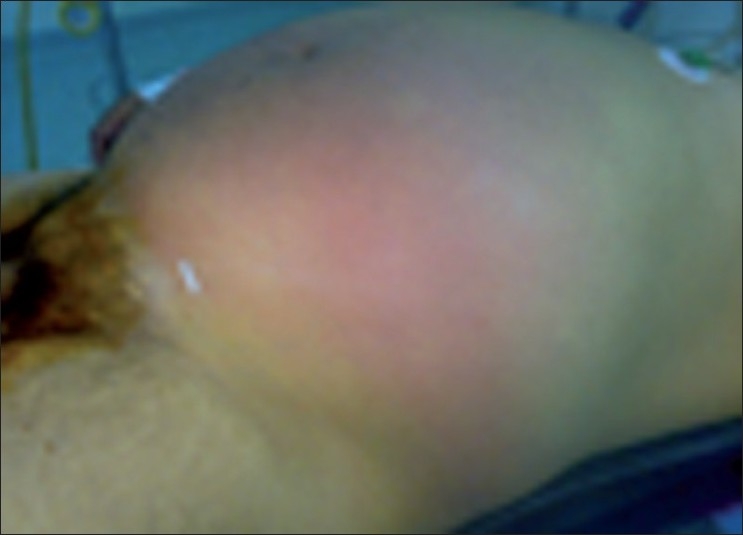
Skin erythema of the abdominal walls

**Figure 2:**
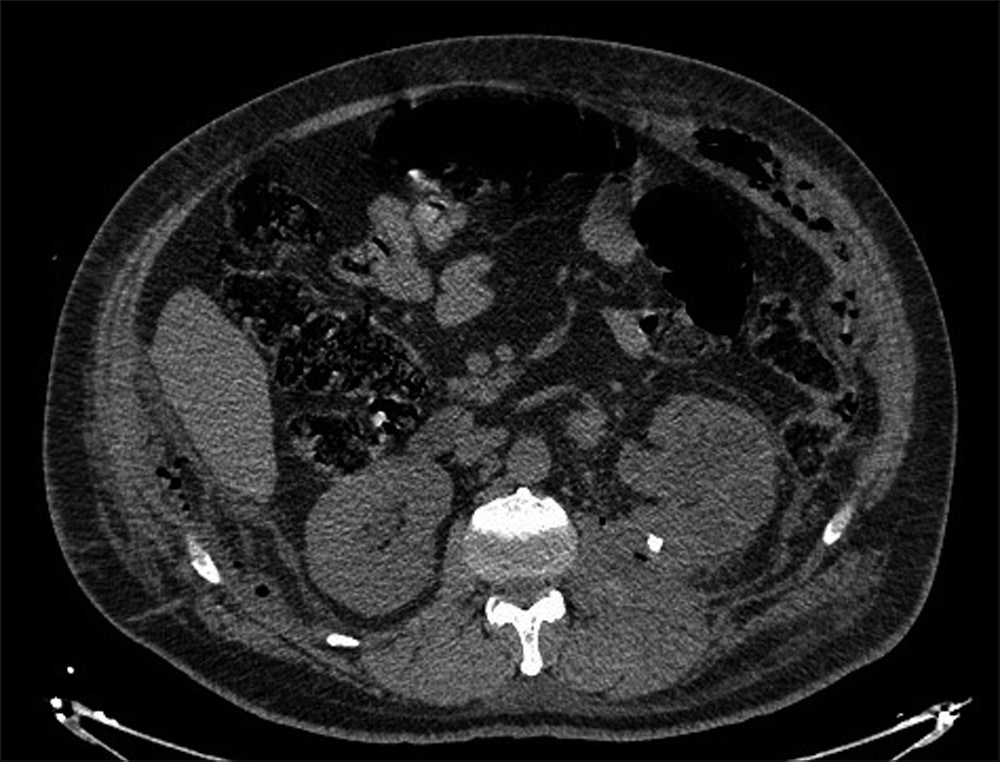
Computed tomography showed left pyonephrosis with middle caliceal stones and perinephric phat infiltration

**Figure 3:**
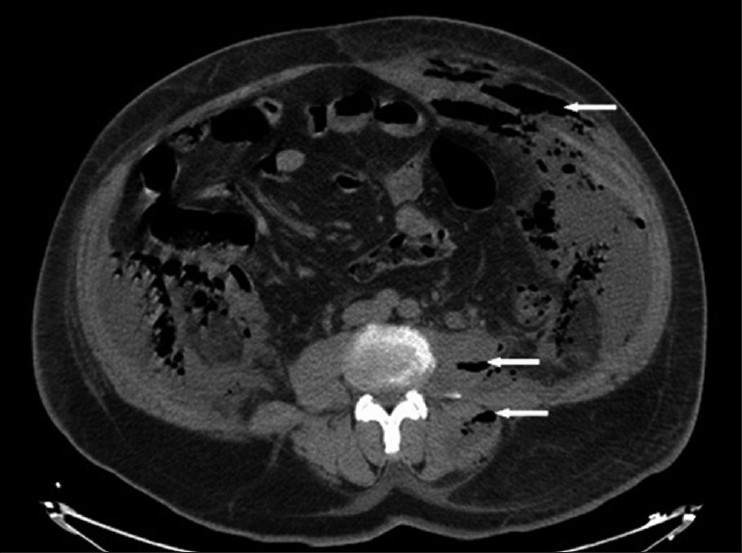
Computed tomography showed scattered gas collection (arrow) over the left psoas, latero-vertebral muscles, fascias, and subcutaneous lower abdominal tissues

Sir,

Retroperitoneal-necrotizing fasciitis is rare, fulminant, and potentially fatal complication of intra-abdominal suppuration. The retroperitoneal origin is very rare. We present the case of a 55-year-old man presented with fever and abdominal pain as chief complaint, who was admitted to the emergency department. He had neither past medical history nor he complaint about urinary symptoms. On investigation, the vital signs were within the normal limits with temperature of 38.5 °C. The abdominal examination was notable for skin erythema [Figure 1] and palpable crepitance without peritoneal signs. The main laboratory finding on admission were leukocytes (19 200 mm^3^), creatinine 1.8 mg/dl, glucose 120 mg/dl, and hyperleucocyturia. Abdominal ultrasonography revealed infiltration of subcutaneous tissue from the left flank, and the visceral exploration was obstructed with bowel gaseous distension. Computed tomography (CT) scanning is then performed, which showed left pyonephrosis [Figure 2] with caliceal stones and scattered gas collection over the left psoas, latero-vertebral muscles, fascias, and subcutaneous lower abdominal tissues [Figure 3] without intraperitoneal remarkable lesion.

**Figure 4:**
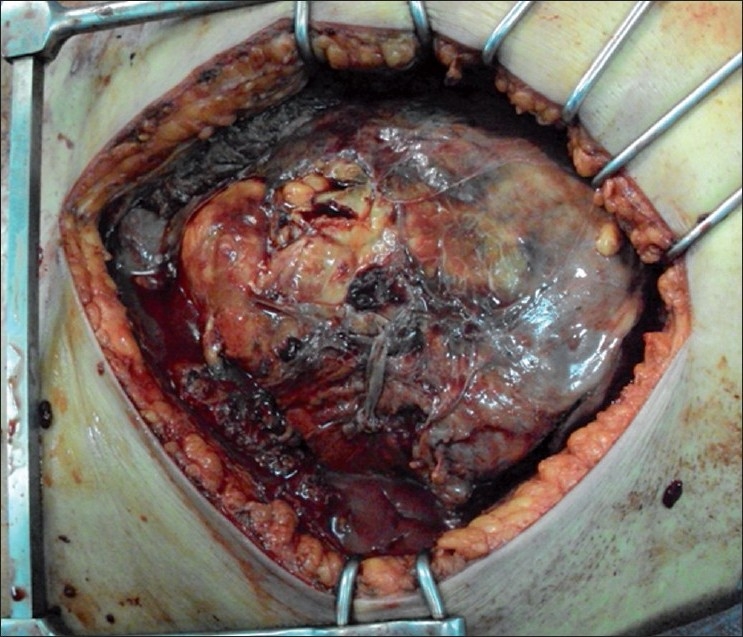
Necrotizing fasciitis involving all fascial and muscle layers of the anterior lower abdomen, left flank, and left retroperitoneum

Under antibiotic coverage, urgent lumbotomy revealed perirenal abscess extended to psoas muscle. Through debridement and surgical nephrostomy was realized. Further iliaque incision [Figure 4] revealed necrotizing fasciitis involving all fascial and muscle layers of the anterior lower abdomen, left flank, and left retroperitoneum. Urine culture grew *Escherichia coli*. The patient was admitted to the intensive care unit after surgery, requiring artificial respiration and monitoring of vital organ function. High dose of antibiotics and dexamethasone were administrated. Aggressive fluid resuscitation and vasoactive drugs where then started. In spite of these means, sepsis rapidly progressed and the patient died four days later.

Retroperitoneal necrotizing fasciitis (RNF) is rapidly progressive and extensive necrosis of subcutaneous fat and fascia caused by retroperitoneal infection.[1] Only three cases associated with renal infection have been described earlier in the literature. A factor predisposing to necrotizing fasciitis (NF) is any state of immunocompromise.[2] The diagnosis of NF is a clinical one. Any single laboratory parameter or radiological finding can suggest but not confirm the diagnosis. Radiological findings suggestive of NF are areas of low echo in ultrasound scan, suggesting liquefaction and hypodense dispersed structures in the subcutaneous tissue or underlying muscle, but without abscess formation on CT; and this finding usually presents at a later stage of the disease process and its absence does not exclude the diagnosis.[3]

The classical entity of gas gangrene is known as a clostridial infection, but in recent reviews most cases of gas forming fasciitis have been due to other organisms, including *Staphylococcus, Group A Streptococcus, Actinomyces, Aerobacter aerogenes, Proteus, Klebsiella,* and *Pseudomonas*.[1,2,4]

We write this letter to reiterate the importance of verifying urinary tract and retroperitoneal organs in front of the suspicion of abdominal necrotizing fasciitis. Early recognition, etiological treatment, broad spectrum antibiotic in combination with an aminoglycoside, imipenem, or metronidazole, aggressive surgical debridement and optimal oxygenation of the infected tissues are the most important principles for management of this potentially lethal disease.[5]
